# AromaDeg, a novel database for phylogenomics of aerobic bacterial degradation of aromatics

**DOI:** 10.1093/database/bau118

**Published:** 2014-12-01

**Authors:** Márcia Duarte, Ruy Jauregui, Ramiro Vilchez-Vargas, Howard Junca, Dietmar H. Pieper

**Affiliations:** ^1^Microbial Interactions and Processes Research Group, HZI—Helmholtz Centre for Infection Research, Inhoffenstr. 7, D-38124 Braunschweig, Germany, ^2^Research Group Microbial Ecology, Metabolism, Genomics and Evolution of Communities of Environmental Microorganisms, CorpoGen. Carrera 5 No. 66A-35, Bogotá, Colombia and ^3^Faculty of Basic and Applied Sciences, Universidad Militar Nueva Granada—UMNG, Campus Cajicá, Bogotá DC, Colombia

## Abstract

Understanding prokaryotic transformation of recalcitrant pollutants and the *in-situ* metabolic nets require the integration of massive amounts of biological data. Decades of biochemical studies together with novel next-generation sequencing data have exponentially increased information on aerobic aromatic degradation pathways. However, the majority of protein sequences in public databases have not been experimentally characterized and homology-based methods are still the most routinely used approach to assign protein function, allowing the propagation of misannotations. AromaDeg is a web-based resource targeting aerobic degradation of aromatics that comprises recently updated (September 2013) and manually curated databases constructed based on a phylogenomic approach. Grounded in phylogenetic analyses of protein sequences of key catabolic protein families and of proteins of documented function, AromaDeg allows query and data mining of novel genomic, metagenomic or metatranscriptomic data sets. Essentially, each query sequence that match a given protein family of AromaDeg is associated to a specific cluster of a given phylogenetic tree and further function annotation and/or substrate specificity may be inferred from the neighboring cluster members with experimentally validated function. This allows a detailed characterization of individual protein superfamilies as well as high-throughput functional classifications. Thus, AromaDeg addresses the deficiencies of homology-based protein function prediction, combining phylogenetic tree construction and integration of experimental data to obtain more accurate annotations of new biological data related to aerobic aromatic biodegradation pathways. We pursue in future the expansion of AromaDeg to other enzyme families involved in aromatic degradation and its regular update.

**Database URL:** http://aromadeg.siona.helmholtz-hzi.de

## Introduction

### Aromatic hydrocarbon: the value of biodegradation

Microorganisms, mostly bacteria, play an important role in the cleanup of contaminated sites as they have acquired the ability to degrade an impressive variety of such aromatic hydrocarbon structures, using them as carbon and energy sources (1). Accordingly, the knowledge on anaerobic bacterial degradation of aromatics is constantly growing (2). However, compared with the anaerobic degradation, aerobic bacterial degradation of aromatic compounds has been much more intensively studied (1). The activation of the aromatic ring commonly proceeds by Rieske non-heme iron oxygenases (3), flavoprotein monooxygenases (4) or soluble diiron monooxygenases (5). Alternatively, activation is mediated by CoA ligases and the formed CoA derivatives are subject to oxygenations. The further aerobic degradation of di- or trihydroxylated intermediates can be catalyzed by either intradiol or extradiol dioxygenases. Contrarly to the superfamily of intradiol dioxygenases, in which all described members belong to the same superfamily, the enzymes reported to be involved in the extradiol ring cleavage of hydroxylated aromatics can be categorized in three different superfamilies: type I extradiol dioxygenases (e.g. catechol 2,3-dioxygenases), which belong to the vicinal oxygen chelate superfamily (6), type II or LigB superfamily extradiol dioxygenases which comprises, among others, protocatechuate 4,5-dioxygenases (7) and type III enzymes such as gentisate dioxygenases, comprising enzymes of the cupin superfamily (8).

### Genomic data resources: living on a log scale

Alongside with the advent and fast upgrading of next-generation sequencing technologies, metagenomics and genome-wide studies, there was an exponential increase in nucleotide and amino acid sequence data (9). However, despite this overall increasing knowledge, also in aerobic aromatic degradation pathways, it is a matter of fact that this information is not clearly structured. The majority of protein sequences in public databases have not been experimentally characterized and homology-based methods are the most routinely used approach to assign and annotate protein function in sequenced genomes and metagenomes. However, the lack of a relatively high sequence identity dismisses an accurate functional assignment and leads to a large number of cumulative homology-based misannotations, which can spread through functional databases (10). In fact, misannotation in enzyme superfamilies containing multiple families that catalyze different reactions is indeed a larger problem of public databases that has been recognized (11) and protein data associated with the aromatic catabolic routes are also significantly affected by this setback (12). Taking into consideration that one crucial goal of environmental biotechnologies is to better understand the potential of microbial communities for the degradation of aromatic pollutants and to explore technologies to restore polluted environments including a systems biology approach, it is crucial to have in hand curated databases, including catabolic key proteins.

### Currently available biodegradation databases

Decades of biochemical studies have produced a considerable wealth of knowledge on biodegradation, and this has started to be categorized and stored in structured databases, which have been valuable for managing biodegradation-related data. For instance, the Biodegrative Strain Database (13) lists degradative bacteria and the hazardous substrates they degrade, including the corresponding literature citations, relevant patents and links to additional biological or chemical data. The University of Minnesota Biocatalysis/Biodegradation Database (UM-BBD) (14) aims to predict plausible pathways for degradation of organic compounds based on known reactions of described microorganisms. However, a careful curation is missing in some of the pathways, such as that for 2,4-dichlorophenoxyacetate, where a 2-chlorodienelactone isomerase is still proposed to be involved (15), a reaction already refuted in 1990 (16), and important pathways are missing such as the mineralization of chlorobenzene via extradiol cleavage of intermediary 3-chlorocatechol (17). Metarouter (18) is focused on the biochemical aspects of biodegradation and allows the analysis of valuable features such as the query of pathways or networks through prediction of chemical biodegradability. However, the absence of information at the sequence level of proteins limits its use for the analysis and annotation of omic data. FunGene (http://fungene.cme.msu.edu/) (19) gives sequence information on some key proteins of biodegradation including direct links to references, however, the database takes over automatic annotations previously given and, thus, does not shed light on probable misannotations. Also Metarouter and other databases, such as the Database of Biodegradative Oxygenases—OxDBase (20) and the web-based server PathPred (21), link the entries to external databases which often direct to proteins electronically annotated in the course of complete genome sequencing projects. As an alternative, Bionemo (22) provides an update to UM-BBD, in which accurate associations between proteins and reactions are based on customized database searches, extensive literature mining and manual curation. Bionemo combines metabolic, genetic and regulatory information but unfortunately its web interface is not updated since 2008.

So far, there is no single resource that provides a direct query based on protein sequence information and data mining through a phylogenomic approach. We assumed that a web interface based on a database that consolidates all these gaps would be an invaluable tool for academic and industrial researchers as well as environmental engineers.

## Construction and content

### General concept: a phylogenomic approach

One of the fundamental paradigms in computational biology is functional prediction by homology (23). Generally, these predictions do not take in account that evolution not only conserves function, but it also generates new functions where the basic biochemical mechanism may be conserved, while the substrate or ligand specificity changes.

Phylogenomic analysis—combining phylogenetic tree construction and the integration of experimental data has been proposed to address these errors and improve functional classification accuracy (23). Molecular phylogenetic analysis has been used for decades for the elucidation of species relationships (24), and the importance of such analysis became more evident once Eisen (25) showed how phylogenomic analysis addresses the deficiencies of function prediction by homology and improves the accuracy (26). Since then, phylogenomic inference of protein molecular function has been applied to the detailed analyses of individual protein families (27), in comparative genomics (28) or for whole-genome analysis (29).

As previous studies have shown that a phylogenomic approach to protein functional classification results in fewer false-positive results, when compared with the pairwise methods of functional classification (26, 30) some databases such as the Lipase Engineering Databases (31), the database of epoxide hydrolases and haloalkane dehalogenases (32) or the carbohydrate-active enzymes database (CAZy) (33) have recognized and explored the relevance of phylogenomics to classify enzymes into subfamilies and to clarify the relationship between protein sequence, structure and substrate specificity.

Also the PFAM database aims to facilitate functional annotation, which here is based on domain assignments (34). This database consists of protein domain families, which are automatically classified on the basis of sequence similarities, where composition and size of the PFAM families vary significantly (34). In case of Rieske non-heme iron oxygenases, as an example, pfam00848 comprises all alpha-subunits discussed here and does not give fine-tuned information. More fine-tuned information can be obtained from the conserved domain database (CDD) which provides annotation of protein sequences with the location of conserved domain footprints (35). Actually CDD maintains an active curation effort that aims at providing classifications for major and well-characterized protein domain families, for example cd03469, the Rieske non-heme iron oxygenase family (35). However, the phylogenomic approach described here is useful for an even more detailed analysis of individual protein families crucial for the degradation of aromatics, as well as for a high-throughput functional classification at a genomic scale.

Thus, to fulfill the existing gap in the available biodegradation databases, we report here an up-to-date and manually curated database with an associated query system that exploits a phylogenomic analysis of aerobic degradation of aromatic compounds—AromaDeg. Through phylogenetic tree construction and integration of experimental data, AromaDeg addresses systematic errors produced by standard methods of protein function prediction and improves the accuracy of functional classification of key proteins of aromatic degradation.

### Contents and curation

In aerobic aromatic degradation, a broad range of peripheral reactions transforms a huge variety of compounds to a restricted set of central intermediates, which are subject to ring-cleavage and subsequent funnelling into the Krebs cycle. Altogether, the catalog of oxygenases involved in activation and cleavage of the aromatic ring is extensive and phylogenetically diverse, including several different families that have been recently compiled for phylogenomic studies (12).

**Figure 1.**
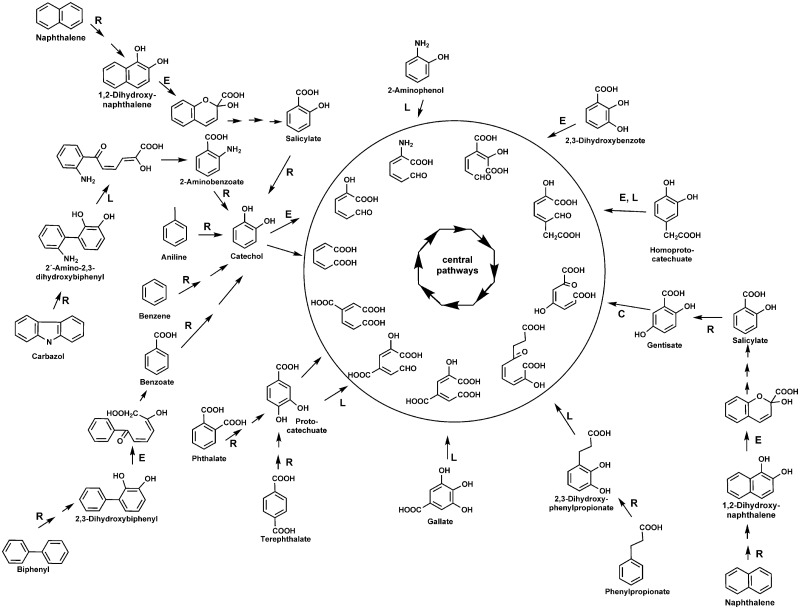
Aerobic metabolism of selected aromatics via di- or trihydroxylated intermediates. Reactions catalyzed by Rieske non-heme iron oxygenases are indicated by R, those catalyzed by extradiol dioxygenases of the vicinal chelate superfamily by an E, those catalyzed by enzymes of the LigB superfamily by an L and those catalyzed by enzymes of the cupin superfamily by a C. Ring-cleavage products are channeled to the Krebs cycle via central reactions.

To avoid misleading annotations and to survey the metabolic potential of aerobic microbial communities, we developed and manually curated a database (with a total of 3605 protein sequences)—AromaDeg, focused on the following key catabolic enzymes for aromatic degradation: α-subunits of Rieske non-heme iron oxygenases, extradiol dioxygenases of the vicinal chelate superfamily, extradiol dioxygenases of the LigB superfamily and extradiol dioxygenases of the cupin superfamily (Figure 1).

#### Rieske non-heme iron oxygenases

The Rieske non-heme iron oxygenases are one of the key enzymes important for aerobic activation and thus degradation of aromatics such as benzoate, benzene, toluene, phthalate, naphthalene or biphenyl (3) (see Figure 1, reactions indicated by R). Members of this superfamily also catalyze monooxygenations, such as salicylate 1- or salicylate 5-hydroxylases or demethylations, such as vanillate *O*-demethylases. It is known that the catalytic components (α-subunits) of all multicomponent Rieske non-heme iron oxygenases are related to each other and several publications attempted their classification. Nakatsu *et al.* (36) pointed out that all Rieske non-heme iron oxygenases devoid of β-subunits (e.g. phthalate 4,5-dioxygenase, vanillate demethylase and carbazol dioxygenase) are related to each other and form a distinct lineage of the superfamily (termed the phthalate oxygenase lineage) and Werlen *et al.* (37) identified four families (naphthalene, toluene/benzene, biphenyl and benzoate/toluate) in a second lineage. In general, the clustering of oxygenases into families correlates with the native substrates oxidized by their members (3). Previous phylogenomic analyses of the α-subunits of Rieske non-heme iron oxygenases (12) also identified two distinct lineages that contain proteins of validated function and correspond to the two mentioned lineages. Following this categorization, we updated these two lineages with sequence data retrieved from the NCBI website, as available in September 2013. This update was performed, as described below in the chapter ‘*Building phylogenetic trees and clusters.*’ Out of the phthalate oxygenase lineage, only enzymes related to those of documented function in the activation of the aromatic ring were further considered for the database and the second lineage can now be differentiated into three families—biphenyl oxygenases, benzoate oxygenases and salicylate oxygenases.

#### Extradiol dioxygenases of the vicinal oxygen chelate superfamily

The extradiol ring-cleavage of catechol is typically catalyzed by type I extradiol dioxygenase*s* (*EXDO*) of the vicinal oxygen chelate superfamily (6). The EXDO I family comprises enzymes that catalyze the dioxygenolytic ring-fission of catecholic derivatives in several bacterial mono- and polyaromatic biodegradation pathways (38) and catalyze the extradiol-cleavage of catechol, 2,3-dihydroxybiphenyl, 1,2-dihydroxynaphthalene, homoprotocatechuate, 2,3-dihydroxy-*p*-cumate, 2,3-dihydroxybenzoate and 7-oxo-11,12-dihydroxydehydroabietate among others (see Figure 1, reactions indicated by E).

Phylogenomic analysis of the deduced protein sequences of EXDO I proteins encoded in the genomes of bacteria having been sequenced until August 2008 showed the presence of three major evolutionary lineages (12). One of these lineages (Lineage 1) comprises nearly all EXDO I proteins of validated function. Lineage 2 contains BphC6 of *Rhodococcus jostii* RHA1 (GenBank: ABO34703) and other previously characterized so-called one-domain extradiol dioxygenases such as BphC2 and BphC3 from *Rhodococcus globerulus* P6 with reported activity against 2,3-dihydroxybiphenyl (39) (subfamily I.1 as defined by Eltis and Bolin (38)), however, most proteins of this lineage have not been characterized thus far. Lineage 3 comprises only a few validated extradiol dioxygenases such as those involved in the turnover of (chloro)benzoquinols and (chloro)hydroxybenzoquinols (LinE chlorobenzoquinol/benzoquinol 1,2-dioxygenases (40) and PcpA 2,6-dichlorobenzoquinol 1,2-dioxygenases (41)). Among the three described lineages, AromaDeg is dedicated to Lineage 1, which comprises most of the EXDO I proteins of validated function. Analysis of the evolutionary relationships among those (38) had shown that they could be differentiated into enzymes having a preference for monocyclic substrates (termed family I.2), and those with a preference for bicyclic substrates (termed family I.3) beside above-mentioned one-domain extradiol dioxygenases (termed family I.1). Since then, an enormous amount of information has been generated (42) and more recent surveys on the phylogeny of extradiol dioxygenases (12), together with the phylogenomic analysis done here, showed that families I.2 and I.3 (38) still form groups supported by high bootstrap values. In addition, Lineage I of type I extradiol dioxygenases comprises various enzymes using miscellaneous substrates such as 2,3-dihydroxybenzoate, which were separately analyzed (family EXDO, miscellaneous substrates).

#### Extradiol dioxygenases of the LigB superfamily

A second superfamily of extradiol dioxygenases is the LigB-type extradiol dioxygenases, members of which are well established as being responsible for the degradation of protocatechuate via the protocatechuate 4,5-dioxygenase pathway (7). Phylogenomic analysis of the deduced protein sequences of LigB-type proteins in 2008 (12) allowed the identification of two families: the protocatechuate and the homoprotocatechuate family, which could now (as in September 2013) be verified with novel proteins mainly identified from genome sequencing projects. The protocatechuate family comprises the protocatechuate 4,5-dioxygenase β-subunits, gallate dioxygenases (43), 2,3-dihydroxyphenylpropionate dioxygenases (44) and additional LigB-type enzymes that have been described to be involved in the degradation of methylgallate (45), of bi- and polycyclic aromatics (46) or of carbazol (47) (see Figure 1, reactions indicated by L).

The homoprotocatechuate family of the LigB superfamily comprises the proteobacterial homoprotocatechuate 2,3-dioxygenases as the one described in *Escherichia coli* (48), the aminophenol 1,6-dioxygenases (see Figure 1, reactions indicated by L) with the β-subunits containing the active site (49) and several other LigB-type extradiol dioxygenases of unknown function, mainly observed in Clostridia and Archaea.

#### Extradiol dioxygenases of the cupin superfamily

Several extradiol dioxygenases of aromatic degradation pathways have been described to belong to the cupin superfamily (8) sharing a common architecture and including key enzymes such as homogentisate 1,2-dioxygenases (50) and 3-hydroxyanthranilate 3,4-dioxygenase (51). Although the above-described enzymes are involved in the degradation of amino acids by various bacteria, gentisate 1,2-dioxygenase is a ring cleavage enzyme involved in the degradation of salicylate or 3-hydroxybenzoate, among other aromatics (52) and thus, has been reported to be involved in the degradation of environmental pollutants such as naphthalene or dibenzofuran (53, 54). Although certain soil organisms, particularly of the genera *Pseudomonas* and *Ralstonia*, have received some attention with regards to the gentisate pathway (52), the importance of this EXDO superfamily for bioremediation as well as its distribution in environmental samples has thus far been neglected. Thus, AromaDeg is focused on the gentisate 1,2-dioxygenase family of the cupin superfamily, including 1-hydroxy-2-naphthoate dioxygenases, reported to be involved in the degradation of polycyclic aromatics such as phenanthrene (55) (see Figure 1, reaction indicated by C).

### Building phylogenetic trees and clusters

Protein sequences of validly described members of the different catabolic protein families mentioned above had been collected and used independently as seeds for searches using the *BLASTP* algorithm (56) against non-redundant protein and environmental sequence databases at GenBank (10) typically with a stringent *E*-value threshold of 1e−20. Protein sequences of representatives of these protein families were then used as seeds for GenBank searches to cover the full range of sequences currently available (by September 2013). All protein sequences were then aligned with MAFFT (57) using default values and phylogenetic trees were constructed with MEGA5 (58) using the neighbor-joining algorithm (59) with *p*-distance correction and pairwise deletion of gaps and missing data. A total of 100 bootstrap replications were done to test for branch robustness.

All proteins of the respective protein family were manually checked for representatives of validated function or for gene clusters encoding that protein and comprising proteins of documented function. Due to currently biased sequencing efforts the generated trees contained various branches with identical or nearly identical proteins of the same function derived from representatives of the same species. To uphold the clearness of these trees, some redundant protein sequences (typically >95–99% of sequence identity) that were obviously over-represented in number were removed. Proteins evidently outgrouping from the constructed phylogenetic trees and belonging to other protein families were eliminated. Phylogenetic trees were inspected for evident branches supported by bootstrap analysis and for proteins of documented function as described above. Clusters were defined as branches supported by bootstrap analysis and a probable function was assigned to its members, if the cluster contains members of validated function.

**Figure 2.**
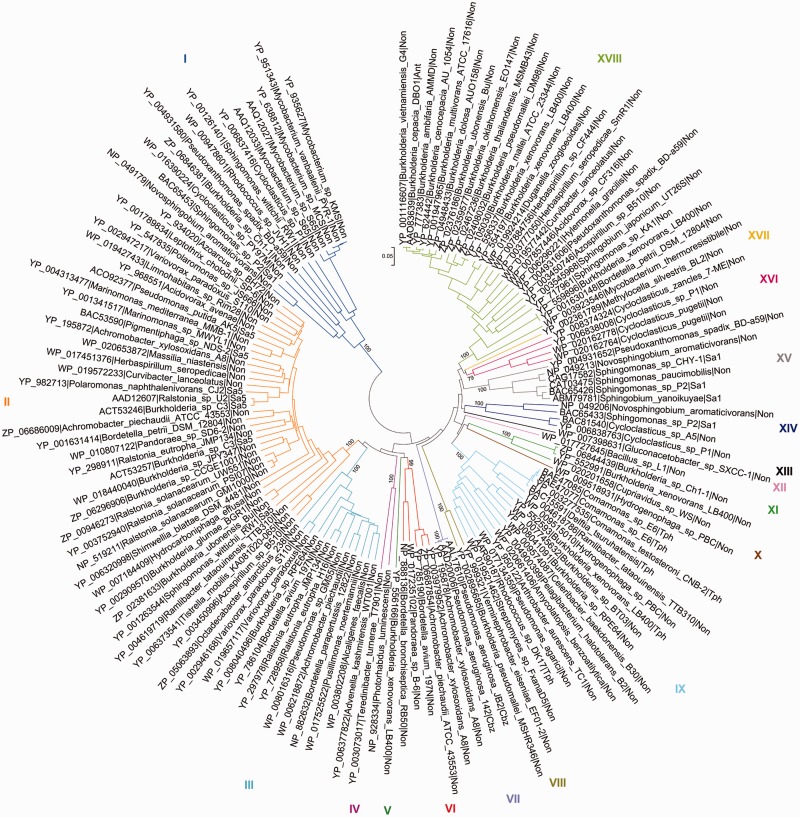
Evolutionary relationships of α-subunits of the salicylate family of Rieske non-heme iron oxygenases. Protein sequences were aligned with MAFFT and the phylogenetic tree was constructed with MEGA5 using the neighbor-joining algorithm with *p*-distance correction and pairwise deletion of gaps and missing data. A total of 100 bootstrap replications were done to test for branch robustness (bootstrap values are shown adjacent to each cluster node) and redundant protein sequences (>95–99% of sequence identity) were removed. According to the documented substrate specificity of representative members they can be clustered as follows: Clusters I, XIV and XV—salicylate 1-hydroxylases; Cluster II—salicylate 5-hydroxylases; Clusters III, IV, V and VI—Rieske oxygenases related to salicylate 5-hydroxylases; Cluster VII—chlorobenzoate dioxygenases; Clusters VIII, X, XI and XII—Rieske oxygenases related to terephthalate dioxygenases; Cluster IX—terephthalate dioxygenases; Cluster XVI—probable salicylate 1-hydroxylases; Cluster XVII—anthranilate dioxygenases of *Burkholderia* and some other organisms. Further information about each cluster is included in Table 1.

As an example, the salicylate family of Rieske non-heme iron oxygenases (see also Pfam domain CD0880) of AromaDeg shown in Figure 2 contains 137 protein sequences that were sorted into 18 clusters supported by high bootstrap values and comprises enzymes capable of transforming *ortho*- and *para*-substituted benzoates. The phylogenetic tree constructed using the maximum-likelihood algorithm showed an identical topology, however, this method suffered from a low speed.

All proteins previously characterized as anthranilate, 2-chlorobenzoate or terephthalate dioxygenases group together in one cluster each (Clusters XVII, VII and IX, respectively), where it can be assumed that all proteins of the respective cluster perform the same reaction (Table 1). Similarly, phylogenomic analysis revealed that all proteins characterized thus far as salicylate 5-hydroxylases group together in Cluster II of the salicylate family of Rieske non-heme iron oxygenases whereas salicylate 1-hydroxylases could be localized in three distinct clusters (Figure 2 and Table 1).

**Table 1.** Phylogenomic clusters of α-subunits of the salicylate family of Rieske non-heme iron oxygenases

Clusters	Representative sequence	Annotation	Substrate	Abbreviation	Pubmed id
I	BAC65453 *Sphingomonas* sp. P2	Salicylate 1-hydroxylases	Salicylate	Sa1	12565867
II	AAD12607 *Ralstonia* sp. U2	Salicylate 5-hydroxylases	Salicylate	Sa5	9573207 22936930 16461653 23266498 21369832
III	ZP_06687231 *Achromobacter piechaudii*	Rieske oxygenases related to salicylate 5-hydroxylases	Unknown	Non	
IV	NP_928334 *Photorhabdus luminescens*	Rieske oxygenases related to salicylate 5-hydroxylases	Unknown	Non	
V	YP_556166 *Burkholderia xenovorans* LB400	Rieske oxygenases related to salicylate 5-hydroxylases	Unknown	Non	
VI	ZP_06687854 *Achromobacter piechaudii*	Rieske oxygenases related to salicylate 5-hydroxylases	Unknown	Non	
VII	AAL17610 *Pseudomonas aeruginosa* JB2	Chlorobenzoate dioxygenases	Chlorobenzoate	Cbz	11722919 10224014
VIII	YP_999321 *Verminephrobacter eiseniae*	Rieske oxygenases related to terephthalate dioxygenases	Unknown	Non	
IX	BAE47077 *Comamonas* sp. E6	Terephthalate dioxygenases	Terephthalate	Tph	16517628 12670689 19734336 16517628 22889862 16181748
X	WP_009518931 *Hydrogenophaga* sp. PBC	Rieske oxygenases related to terephthalate dioxygenases	Unknown	Non	
XI	YP_552991 *Burkholderia xenovorans* LB400	Rieske oxygenases related to terephthalate dioxygenases	Unknown	Non	
XII	WP_017727645 *Bacillus* sp. L1	Rieske oxygenases related to terephthalate dioxygenases	Unknown	Non	
XIII	WP_007398631 *Gluconacetobacter* sp. SXCC-1	Rieske oxygenases related to salicylate 1-hydroxylases	Unknown	Non	
XIV	BAC65433 *Sphingomonas* sp. P2	Salicylate 1-hydroxylases	Salicylate	Sa1	12565867
XV	BAC65426 *Sphingomonas* sp. P2	Salicylate 1-hydroxylases	Salicylate	Sa1	12565867 15649397 15528538
XVI	YP_008374324 *Cycloclasticus zancles* 7 ME	Probable salicylate 1-hydroxylases	Probably salicylate	Non	
XVII	YP_002361789 *Methylocella silvestris* BL2	Probable salicylate 1-hydroxylases	Probably salicylate	Non	
XVIII	AAO83639 *Burkholderia cepacia*	Anthranilate dioxygenases of *Burkholderia* and some other organisms	Anthranilate	Ant	13129960

*Note*s. List of the three- or four-letter code indicate the experimentally validated function and/or substrate: Ant, anthranilate; Cbz, chlorobenzoate; Sa1, salicylate (salicylate 1-hydroxylases); Sa5, salicylate (salicylate 5-hydroxylases); Tph, terephthalate.

All other nine phylogenetic trees—namely, those showing the α-subunits of the phthalate, biphenyl and benzoate family of Rieske non-heme iron oxygenases; those showing families I.2, I.3 (38) and a third family of extradiol dioxygenases of the vicinal oxygen chelate superfamily; those showing the homoprotocatechuate and protocatechuate families of the LigB superfamily of extradiol dioxygenases and a tree showing the gentisate family of the cupin extradiol dioxygenases—and the cluster descriptions of the AromaDeg database are summarized and available in the Supplementary Material (Supplementary Figures S1–S9 and Supplementary Tables S1–S9).

## The usefulness of the database

Blast searches (http://blast.ncbi.nlm.nih.gov/Blast.cgi) using proteins closely related to those of validated function often show highest similarity to enzymes with a proper annotation. As an example, high similarity of the protein with the GenBank accession number YP_004931652 from *Pseudoxanthomonas spadix* BD-a59 (*E*-values down to 0) was observed with enzymes annotated as salicylate 5-hydroxylases, however, also with chlorobenzoate dioxygenases such as the enzyme indentified from *Pseudomonas aeruginosa* 142 (GenBank accession number AAD20006, *E*-value 10^−^^158^) (60), whereas phylogenetic analysis was capable to clearly separate salicylate 5-hydroxylases and chlorobenzoate dioxygenases (see Figure 1, Clusters II and VII). More importantly, enzymes showing highest similarity to Cluster III enzymes, such as YP_002946168 from *Variovorax paradoxus* S110 (*E*-value of 0), are predominantly annotated as biphenyl 2,3-dioxygenases. The most similar enzymes of validated function are, however, salicylate 5-hydroxylases (e.g. salicylate 5-hydroxylase ACT53246 from *Burkholderia* sp. B2 (*E* = 2 × 10^−^^119^)) (61) and salicylate 1-hydroxylases (*E* = 6 × 10^−^^86^) (62). Our analysis (see Figure 1, Cluster III) clearly shows that cluster III enzymes among enzymes of validated function are most closely related to salicylate 5-hydroxylases, however representatives of this cluster need to be functionally characterized to allow any clear functional assignment.

It can, thus, be summarized that the assignment of probable functions to novel proteins involved in aromatic degradation simply based on similarity using best hits leads to functional misannotations as phylogenetically different enzymes may show equal similarities to the query enzyme. Moreover, a significant amount of misannotations is already available and landmarks of proteins with validated function are hidden behind the massive amount of information available. All these problems may be overcome by the use of AromaDeg.

## The query system: a public and user-friendly web interface

To build up a searchable database with a user-friendly web interface we standardized the data source files by collecting all the compiled protein sequences in a multi-fasta format with headers composed of the protein id (accession number according to GenBank (http://www.ncbi.nlm.nih.gov)), the organism that harbors the given protein and a three- or four-letter code that describes the experimentally validated function and/or substrate. All enzymes that do not have a validated function contain the three-letter code ‘Non’ in their header.

All sequences belonging to each one of the trees are made available as a single file comprising the curated information and the complete sequence set unaligned, or aligned by MAFFT (57). All mentioned source files can be retrieved online, in the *download* section of the website (see link below).

The AromaDeg database can be queried at http://aro madeg.siona.helmholtz-hzi.de and in contrast to most biodegradation-related databases, it does not require a query based on previous knowledge (name of the organism, compound or enzyme) but simply a protein sequence which can be particularly advantageous when mining in novel genomes or high-throughput metagenomic or metatranscriptomic data sets. The web interface allows the query of source files up to 20 Mb. Once submitted, the retrieved information is, in a first step, compared with the entire database using the blastp program (56) with adjustable parameters. In a second step, each query sequence that matches a given protein family of the AromaDeg is aligned with the sequences constituting that protein family using MAFFT (57) with parameters ‘—globalpair—retree 300—maxiterate 300’. The multiple alignment is then used to build a phylogenetic tree using the neighbor-joining algorithm and the Ka/Ks model (63), with bootstrap values calculated to provide branch support (from 25 iterations) as implemented in the Seaview program (64). The tree file produced in Newick format is then used by the Newick Utilities program suite (65) to generate SVG (Support Vector Graphics) images of the final tree and independent clusters, which include the query of the user. Based on this result, it is possible to identify the nearest cluster the candidate sequence belongs to, and thus, obtain information on the probable protein function, substrate specificity or taxonomic classification. For this purpose, manually compiled and curated information about the members of the same cluster is available in the three- or four-letter code of the protein headers (see Figure 2 and Supplementary Figures S1–S9) and provided in the corresponding table (see Table 1 and Supplementary Tables S1–S9). Upon a successful run, the website sends an email to the user with a link where the above-mentioned result files are available for download. More details about the usage and output of AromaDeg can be retrieved online in the *Extended Manual* available for download.

Given the multiplicity of options, AromaDeg allows a flexible use of the manually curated biological data, ranging from an automatized pipeline where the final phylogenetic trees including the queries can be retrieved as output, to a more independent option in which the user can download the curated source files (see download section of the web interface) and perform the phylogenetic analysis using other computational tools.

## Applications and future directions

Resources such as repositories of sequenced genomes and all subject-specific databases are increasingly faced with the challenge of ensuring data accuracy and efficient management and curation. However, these efforts are hampered by factors such as the reliability of curation and the lack of incentives for researchers to contribute, among others. ‘To date, not much of the research community is rolling up its sleeves to annotate’ (9). We wanted to address this issue with AromaDeg, as an effort to compile biological data related to the bacterial aerobic aromatic degradation and initiate a reliable annotation. The compilation of manually curated data has a wide range of applications in the fields of molecular biology and genomics. As a specialized database in aerobic aromatic degradation, it is not only a better-suited tool than non-specialized databases for improving the design of PCR primers and probes targeting a cluster of catabolic enzymes of a given protein family (66, 67), but also specifically for accurate annotation of genomes and high-throughput data generated by different omics approaches. A preliminary version of the biological data compilation of AromaDeg has been efficiently applied for a knowledge-based design of probes for functional gene arrays covering the diversity of aromatic degradation reactions (42) and the annotation of metagenomic sequences (68).

These successful applications have encouraged the recent update of the database and the development of a user-friendly web interface to open the database to the public and to make it suitable for high-throughput applications. To the best of our knowledge, this is the only resource available that, in a fast and precise manner, combines phylogenetic analysis and a fine level of accurate annotation of new biological data related to aerobic aromatic biodegradation pathways.

We aim in future to expand the database to other enzyme families involved in aromatic degradation, including anaerobic key enzymes and to update regularly the already described enzyme families. An upgrade of the database by enzymes of validated function, which may have been overseen in the database construction, is for sure welcome. We therefore expect this tool to be used in a broad spectrum of scientific and applied research. Furthermore, we strongly believe in the robustness of this approach for reliable function prediction and we envision its use in wide range of fields, other than biodegradation, to hopefully allow researchers to dive deeper in the functional properties of enzymes.

## Accessibility

AromaDeg is freely available online for educational and research purposes by non-profit institutions at http://aromadeg.siona.helmholtz-hzi.de. All sequences described in this article can be downloaded from that site.

## Supplementary data

Supplementary data are available at *Database* Online.

Supplementary Data

## References

[1] Pérez-Pantoja D. González B. Pieper D.H. (2010) Aerobic degradation of aromatic hydrocarbons. In: Timmis K.N. (ed). Handbook of Hydrocarbon and Lipid Microbiology. Springer, Berlin, pp. 799–837.

[2] Davidova I.A. Gieg L.M. Duncan K.E. (2007) Anaerobic phenanthrene mineralization by a carboxylating sulfate-reducing bacterial enrichment. ISME J., 1, 436–442. 18043662 10.1038/ismej.2007.48

[3] Gibson D.T. Parales R.E. (2000) Aromatic hydrocarbon dioxygenases in environmental biotechnology. Curr. Opin. Biotechnol., 11, 236–243. 10851146 10.1016/s0958-1669(00)00090-2

[4] van Berkel W.J. Kamerbeek N.M. Fraaije M.W. (2006) Flavoprotein monooxygenases, a diverse class of oxidative biocatalysts. J. Biotechnol., 124, 670–689. 16712999 10.1016/j.jbiotec.2006.03.044

[5] Leahy J.G. Batchelor P.J. Morcomb S.M. (2003) Evolution of the soluble diiron monooxygenases. FEMS Microbiol. Rev., 27, 449–479. 14550940 10.1016/S0168-6445(03)00023-8

[6] Gerlt J.A. Babbitt P.C. (2001) Divergent evolution of enzymatic function: mechanistically diverse superfamilies and functionally distinct suprafamilies. Annu. Rev. Biochem., 70, 209–246. 11395407 10.1146/annurev.biochem.70.1.209

[7] Sugimoto K. Senda T. Aoshima H. (1999) Crystal structure of an aromatic ring opening dioxygenase LigAB, a protocatechuate 4,5-dioxygenase, under aerobic conditions. Structure, 7, 953–965. 10467151 10.1016/s0969-2126(99)80122-1

[8] Dunwell J.M. Khuri S. Gane P.J. (2000) Microbial relatives of the seed storage proteins of higher plants: conservation of structure and diversification of function during evolution of the cupin superfamily. Microbiol. Mol. Biol. Rev., 64, 153–179. 10704478 10.1128/mmbr.64.1.153-179.2000 PMC98990

[9] Howe D. Costanzo M. Fey P. (2008) Big data: the future of biocuration. Nature, 455, 47–50. 18769432 10.1038/455047a PMC2819144

[10] Vilchez-Vargas R. Junca H. Pieper D.H. (2010) Metabolic networks, microbial ecology and ‘omics’ technologies: towards understanding in situ biodegradation processes. Environ. Microbiol., 12, 3089–3104. 20860734 10.1111/j.1462-2920.2010.02340.x

[11] Schnoes A.M. Brown S.D. Dodevski I. (2009) Annotation error in public databases: misannotation of molecular function in enzyme superfamilies. PLoS Comput. Biol., 5, e1000605. 20011109 10.1371/journal.pcbi.1000605 PMC2781113

[12] Pérez-Pantoja D. Donoso R. Junca H. (2009) Phylogenomics of aerobic bacterial degradation of aromatics. In: Timmis K.N. (ed). Handbook of Hydrocarbon and Lipid Microbiology. Springer, Berlin, pp. 1355–1397.

[13] Urbance J.W. (2003) BSD: the biodegradative strain database. Nucleic Acids Res., 31, 152–155. 12519970 10.1093/nar/gkg032 PMC165479

[14] Ellis L.B. Roe D. Wackett L.P. (2006) The University of Minnesota Biocatalysis/Biodegradation Database: the first decade. Nucleic Acids Res., 34, D517–D521. 16381924 10.1093/nar/gkj076 PMC1347439

[15] Schwien U. Schmidt E. Knackmuss H-J. (1988) Degradation of chlorosubstituted aromatic compounds by *Pseudomonas* sp. strain B13: fate of 3,5-dichlorocatechol. Arch. Microbiol., 150, 78–84.

[16] Kuhm A.E. Schlömann M. Knackmuss H.J. (1990) Purification and characterization of dichloromuconate cycloisomerase from *Alcaligenes eutrophus* JMP 134. Biochem. J.*,* 266, 877–883. 2327971 PMC1131220

[17] Mars A.E. Kasberg T. Kaschabek S.R.*.* (1997) Microbial degradation of chloroaromatics: use of the meta-cleavage pathway for mineralization of chlorobenzene. J. Bacteriol., 179, 4530–4537. 9226262 10.1128/jb.179.14.4530-4537.1997 PMC179288

[18] Pazos F. Guijas D. Valencia A. (2005) MetaRouter: bioinformatics for bioremediation. Nucleic Acids Res., 33, D588–D592. 15608267 10.1093/nar/gki068 PMC540022

[19] Fish J.A. Chai B. Wang Q.*.* (2013) FunGene: the functional gene pipeline and repository. Front. Microbiol., 4, 291. 24101916 10.3389/fmicb.2013.00291 PMC3787254

[20] Arora P.K. Kumar M. Chauhan A. (2009) OxDBase: a database of oxygenases involved in biodegradation. BMC Res. Notes, 2, 67. 19405962 10.1186/1756-0500-2-67 PMC2683861

[21] Moriya Y. Shigemizu D. Hattori M.*.* (2010) PathPred: an enzyme-catalyzed metabolic pathway prediction server. Nucleic Acids Res., 38, W138–W143. 20435670 10.1093/nar/gkq318 PMC2896155

[22] Carbajosa G. Trigo A. Valencia A. (2009) Bionemo: molecular information on biodegradation metabolism. Nucleic Acids Res., 37, D598–D602. 18986994 10.1093/nar/gkn864 PMC2686592

[23] Sjolander K. (2004) Phylogenomic inference of protein molecular function: advances and challenges. Bioinformatics, 20, 170–179. 14734307 10.1093/bioinformatics/bth021

[24] Woese C.R. (1987) Bacterial evolution. Microbiol. Rev., 51, 221–271. 2439888 10.1128/mr.51.2.221-271.1987 PMC373105

[25] Eisen J. (1998) Phylogenomics: improving functional predictions for uncharacterized genes by evolutionary analysis. Genome Res., 8, 163–167. 9521918 10.1101/gr.8.3.163

[26] Eisen J.A. Fraser C.M. (2003) Phylogenomics: intersection of evolution and genomics. Science, 300, 1706–1707. 12805538 10.1126/science.1086292

[27] Gadelle D. Filee J. Buhler C. (2003) Phylogenomics of type II DNA topoisomerases. BioEssays, 25, 232–242. 12596227 10.1002/bies.10245

[28] Sicheritz-Pontén T. Andersson S.G.E. (2001) A phylogenomic approach to microbial evolution. Nucleic Acids Res., 29, 545–552. 11139625 10.1093/nar/29.2.545 PMC29656

[29] Eisen J.A. Nelson K.E. Paulsen I.T. (2002) The complete genome sequence of *Chlorobium tepidum* TLS, a photosynthetic, anaerobic, green-sulfur bacterium. Proc. Natl Acad. Sci. U. S. A*.* 99, 9509–9514. 12093901 10.1073/pnas.132181499 PMC123171

[30] Zmasek C. Eddy S.R. (2002) RIO: analyzing proteomes by automated phylogenomics using resampled inference of orthologs. BMC Bioinformatics, 3, 14. 12028595 10.1186/1471-2105-3-14 PMC116988

[31] Fischer M. Pleiss J. (2003) The Lipase Engineering Database: a navigation and analysis tool for protein families. Nucleic Acids Res., 31, 319–321. 12520012 10.1093/nar/gkg015 PMC165462

[32] Barth S. Fischer M. Schmid R.D. (2004) The database of epoxide hydrolases and haloalkane dehalogenases: one structure, many functions. Bioinformatics, 20, 2845–2847. 15117755 10.1093/bioinformatics/bth284

[33] Lombard V. Ramulu H.G. Drula E.*.* (2014) The carbohydrate-active enzymes database (CAZy) in 2013. Nucleic Acids Res., 42, D490–D495. 24270786 10.1093/nar/gkt1178 PMC3965031

[34] Finn R.D. Mistry J. Tate J.*.* (2010) The Pfam protein families database. Nucleic Acids Res., 38, D211–D222, D141. 19920124 10.1093/nar/gkp985 PMC2808889

[35] Marchler-Bauer A. Zheng C. Chitsaz F. (2013) CDD: conserved domains and protein three-dimensional structure. Nucleic Acids Res., 41, D348–DD352. 23197659 10.1093/nar/gks1243 PMC3531192

[36] Nakatsu C.H. Straus N.A. Wyndham R.C. (1995) The nucleotide sequence of the Tn5271 3-chlorobenzoate 3,4-dioxygenase genes (*cba*AB) unites the class IA oxygenases in a single lineage. Microbiology, 141, 485–495. 7704279 10.1099/13500872-141-2-485

[37] Werlen C. Kohler H-P.E. van der Meer J.R. (1996) The broad substrate Cclorobenzene dioxygenase and *cis*-chlorobenzene dihydrodiol dehydrogenase of *Pseudomonas* sp. strain P51 are linked evolutionarily to the enzymes for benzene and toluene degradation. J. Biol. Chem., 271, 4009–4016. 8626733 10.1074/jbc.271.8.4009

[38] Eltis L.D. Bolin J.T. (1996) Evolutionary relationships among extradiol dioxygenases. J. Bacteriol., 178, 5930–5937. 8830689 10.1128/jb.178.20.5930-5937.1996 PMC178449

[39] Asturias J.A. Timmis K.N. (1993) Three different 2,3-dihydroxybiphenyl-1,2-dioxygenase genes in the gram-positive polychlorobiphenyl-degrading bacterium *Rhodococcus globerulus* P6. J. Bacteriol., 175, 4631–4640. 8335622 10.1128/jb.175.15.4631-4640.1993 PMC204914

[40] Miyauchi K. Adachi Y. Nagata Y. (1999) Cloning and sequencing of a novel meta-cleavage dioxygenase gene whose product is involved in degradation of gamma-hexachlorocyclohexane in *Sphingomonas paucimobilis*. J. Bacteriol., 181, 6712–6719. 10542173 10.1128/jb.181.21.6712-6719.1999 PMC94136

[41] Xu L. Resing K. Lawson S.L.*.* (1999) Evidence that *pcp*A encodes 2,6-dichlorohydroquinone dioxygenase, the ring cleavage enzyme required for pentachlorophenol degradation in *Sphingomonas chlorophenolica* strain ATCC 39723. Biochemistry, 38, 7659–7669. 10387005 10.1021/bi990103y

[42] Vilchez-Vargas R. Geffers R. Suarez-Diez M.*.* (2013) Analysis of the microbial gene landscape and transcriptome for aromatic pollutants and alkane degradation using a novel internally calibrated microarray system. Environ. Microbiol., 15, 1016–1039. 22515215 10.1111/j.1462-2920.2012.02752.x

[43] Nogales J. Canales A. Jimenez-Barbero J.*.* (2005) Molecular characterization of the gallate dioxygenase from *Pseudomonas putida* KT2440. The prototype of a new subgroup of extradiol dioxygenases. J. Biol. Chem., 280, 35382–35390. 16030014 10.1074/jbc.M502585200

[44] Diaz E. Ferrandez A. Prieto M.A. (2001) Biodegradation of aromatic compounds by Escherichia coli. Microbiol. Mol. Biol. Rev., 65, 523–569. 11729263 10.1128/MMBR.65.4.523-569.2001 PMC99040

[45] Kasai D. Masai E. Miyauchi K.*.* (2004) Characterization of the 3-O-methylgallate dioxygenase gene and evidence of multiple 3-O-methylgallate catabolic pathways in *Sphingomonas paucimobilis* SYK-6. J. Bacteriol., 186, 4951–4959. 15262932 10.1128/JB.186.15.4951-4959.2004 PMC451629

[46] Laurie A.D. LloydJones G. (1999) Conserved and hybrid meta-cleavage operons from PAH-degrading *Burkholderia* RP007. Biochem. Biophys. Res. Commun., 262, 308–314. 10448110 10.1006/bbrc.1999.1153

[47] Sato S. Ouchiyama N. Kimura T.*.* (1997) Cloning of genes involved in carbazole degradation of *Pseudomonas* sp. strain CA10: nucleotide sequences of genes and characterization of meta-cleavage enzymes and hydrolase. J. Bacteriol., 179, 4841–4849. 9244273 10.1128/jb.179.15.4841-4849.1997 PMC179332

[48] Roper D.I. Cooper R.A. (1990) Subcloning and nucleotide sequence of the 3,4-dihydroxyphenylacetate (homoprotocatechuate) 2,3-dioxygenase gene from *Escherichia coli* C. FEBS Lett., 275, 53–57. 2261999 10.1016/0014-5793(90)81437-s

[49] Takenaka S. Murakami S. Shinke R.*.* (1997) Novel genes encoding 2-aminophenol 1,6-dioxygenase from *Pseudomonas* species AP-3 growing on 2-aminophenol and catalytic properties of the purified enzyme. J. Biol. Chem., 272, 14727–14732. 9169437 10.1074/jbc.272.23.14727

[50] Arias-Barrau E. Olivera E.R. Luengo J.M. (2004) The homogentisate pathway: a central catabolic pathway involved in the degradation of l-phenylalanine, l-tyrosine, and 3-hydroxyphenylacetate in *Pseudomonas putida*. J. Bacteriol., 186, 5062–5077. 15262943 10.1128/JB.186.15.5062-5077.2004 PMC451635

[51] Muraki T. Taki M. Hasegawa Y.*.* (2003) Prokaryotic homologs of the eukaryotic 3-hydroxyanthranilate 3,4-dioxygenase and 2-amino-3-carboxymuconate-6-semialdehyde decarboxylase in the 2-nitrobenzoate degradation pathway of *Pseudomonas fluorescens* strain KU-7. Appl. Environ. Microbiol., 69, 1564–1572. 12620844 10.1128/AEM.69.3.1564-1572.2003 PMC150085

[52] Adams M.A. Singh V.K. Keller B.O. (2006) Structural and biochemical characterization of gentisate 1,2-dioxygenase from *Escherichia coli* O157:H7. Mol. Microbiol., 61, 1469–1484. 16930152 10.1111/j.1365-2958.2006.05334.x

[53] Grund E. Denecke B. Eichenlaub R. (1992) Naphthalene degradation via salicylate and gentisate by *Rhodococcus* sp. strain B4. Appl. Environ. Microbiol., 58, 1874–1877. 1622263 10.1128/aem.58.6.1874-1877.1992 PMC195698

[54] Fortnagel P. Harms H. Wittich R-M.*.* (1990) Metabolism of dibenzofuran by *Pseudomonas* sp. strain HH69 and the mixed culture HH27. Appl. Environ. Microbiol., 56, 1148–1156. 16348159 10.1128/aem.56.4.1148-1156.1990 PMC184358

[55] Iwabuchi T. Harayama S. (1998) Biochemical and molecular characterization of 1-hydroxy-2-naphthoate dioxygenase from *Nocardioides* sp. KP7. J. Biol. Chem., 273, 8332–8336. 9525941 10.1074/jbc.273.14.8332

[56] Altschul S.F. Gish W. Miller W. (1990) Basic local alignment search tool. J. Mol. Biol., 215, 403–410. 2231712 10.1016/S0022-2836(05)80360-2

[57] Katoh K. Standley D.M. (2013) MAFFT multiple sequence alignment software version 7: improvements in performance and usability. Mol. Biol. Evol., 30, 772–780. 23329690 10.1093/molbev/mst010 PMC3603318

[58] Tamura K. Peterson D. Peterson N.*.* (2011) MEGA5: molecular evolutionary genetics analysis using maximum likelihood, evolutionary distance, and maximum parsimony methods. Mol. Biol. Evol., 28, 2731–2739. 21546353 10.1093/molbev/msr121 PMC3203626

[59] Saitou N. Nei M. (1987) The neighbor-joining method: a new method for reconstructing phylogenetic trees. Mol. Biol. Evol., 4, 406–425. 3447015 10.1093/oxfordjournals.molbev.a040454

[60] Tsoi T.V. Plotnikova E.G. Cole J.R.*.* (1999) Cloning, expression, and nucleotide sequence of the *Pseudomonas aeruginosa* 142 *ohb* genes coding for oxygenolytic ortho dehalogenation of halobenzoates. Appl. Environ. Microbiol., 65, 2151–2162. 10224014 10.1128/aem.65.5.2151-2162.1999 PMC91311

[61] Tittabutr P. Cho I.K. Li Q.X. (2011) Phn and Nag-like dioxygenases metabolize polycyclic aromatic hydrocarbons in *Burkholderia* sp. C3. Biodegradation, 22, 1119–1133. 21369832 10.1007/s10532-011-9468-y

[62] Pinyakong O. Habe H. Yoshida T. (2003) Identification of three novel salicylate 1-hydroxylases involved in the phenanthrene degradation of *Sphingobium* sp. strain P2 Biochem. Biophys. Res. Commun., 301, 350–357. 10.1016/s0006-291x(02)03036-x 12565867

[63] Li W.-H. (1993) Unbiased estimation of the rates of synonymous and nonsynonymous substitution. J. Mol. Evol., 36, 96–99. 8433381 10.1007/BF02407308

[64] Gouy M. Guindon S. Gascuel O. (2010) SeaView Version 4: a multiplatform graphical user interface for sequence alignment and phylogenetic tree building. Mol. Biol. Evol., 27, 221–224. 19854763 10.1093/molbev/msp259

[65] Junier T. Zdobnov E.M. (2010) The Newick utilities: high-throughput phylogenetic tree processing in the Unix shell. Bioinformatics, 26, 1669–1670. 20472542 10.1093/bioinformatics/btq243 PMC2887050

[66] Junca H. Pieper D.H. (2004) Functional gene diversity analysis in BTEX contaminated soils by means of PCR-SSCP DNA fingerprinting: comparative diversity assessment against bacterial isolates and PCR-DNA clone libraries. Environ. Microbiol., 6, 95–110. 14756875 10.1046/j.1462-2920.2003.00541.x

[67] Witzig R. Junca H. Hecht H.J. (2006) Assessment of toluene/biphenyl dioxygenase gene diversity in benzene-polluted soils: links between benzene biodegradation and genes similar to those encoding isopropylbenzene dioxygenases. Appl. Environ. Microbiol., 72, 3504–3514. 16672497 10.1128/AEM.72.5.3504-3514.2006 PMC1472391

[68] Guazzaroni M.E. Herbst F.A. Lores I.*.* (2013) Metaproteogenomic insights beyond bacterial response to naphthalene exposure and bio-stimulation. ISME J., 7, 122–136. 22832345 10.1038/ismej.2012.82 PMC3526184

