# Supplementary material for: AromaDeg, a novel database for phylogenomics of aerobic bacterial degradation of aromatics
Source: Database (Oxford). 2014 Dec 1;2014:bau118. doi: 10.1093/database/bau118 (PMC4250580; doi:10.1093/database/bau118)
Supplement: Supplementary Data [file supp_2014_bau118_index.html]

Supplementary Data 

# AromaDeg, a novel database for phylogenomics of aerobic bacterial degradation of aromatics

## Supplementary Data

files

**Files in this Data Supplement:**

- Supplementary Data - zip file
