# Supplementary figures and images for: AromaDeg, a novel database for phylogenomics of aerobic bacterial degradation of aromatics
Source: Database (Oxford). 2014 Dec 1;2014:bau118. doi: 10.1093/database/bau118 (PMC4250580; doi:10.1093/database/bau118)

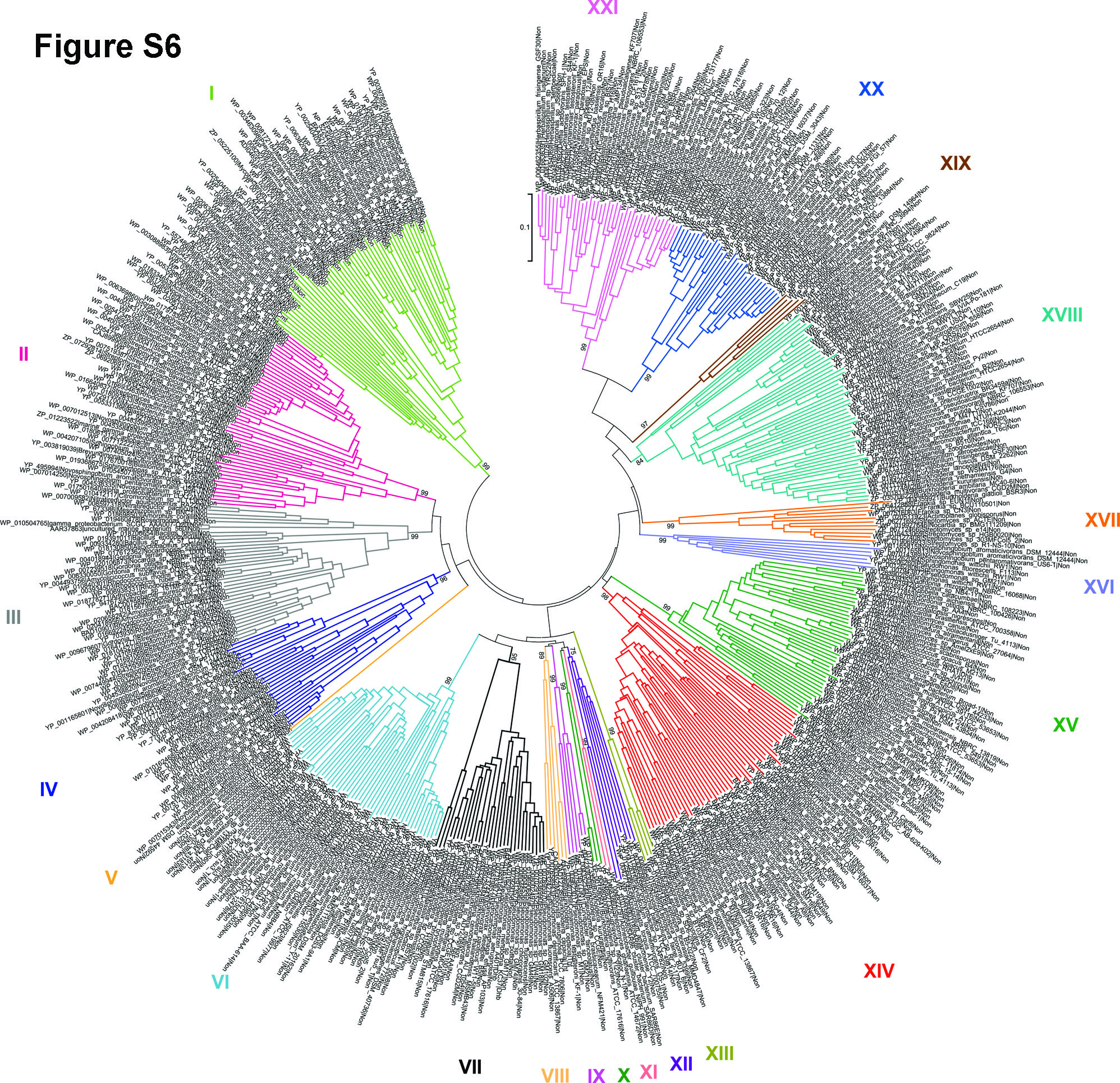

Supplement: Supplementary Data [file supp_bau118_suppl_data.zip › Figure_S6.jpg]

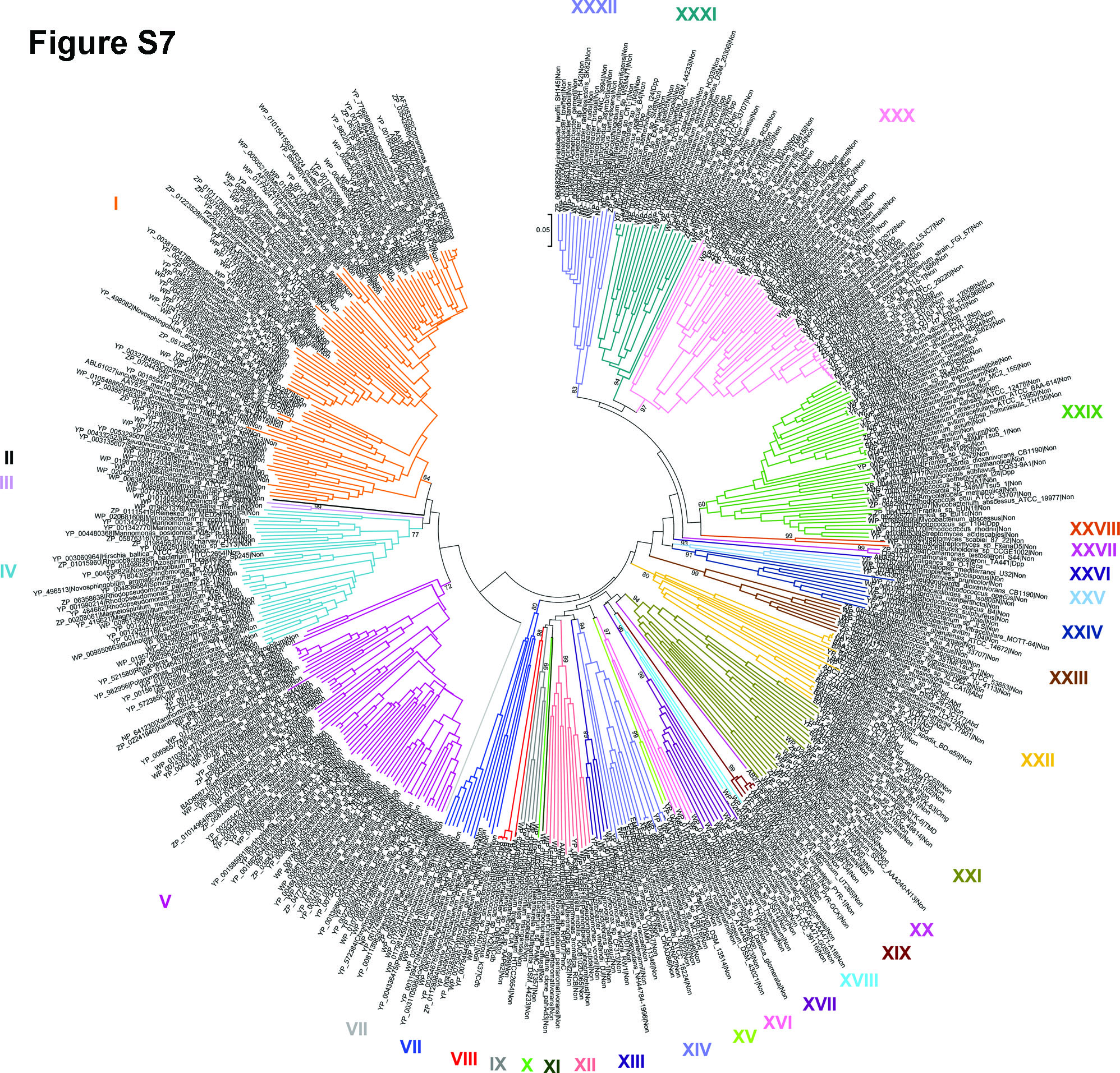

Supplement: Supplementary Data [file supp_bau118_suppl_data.zip › Figure_S7.jpg]

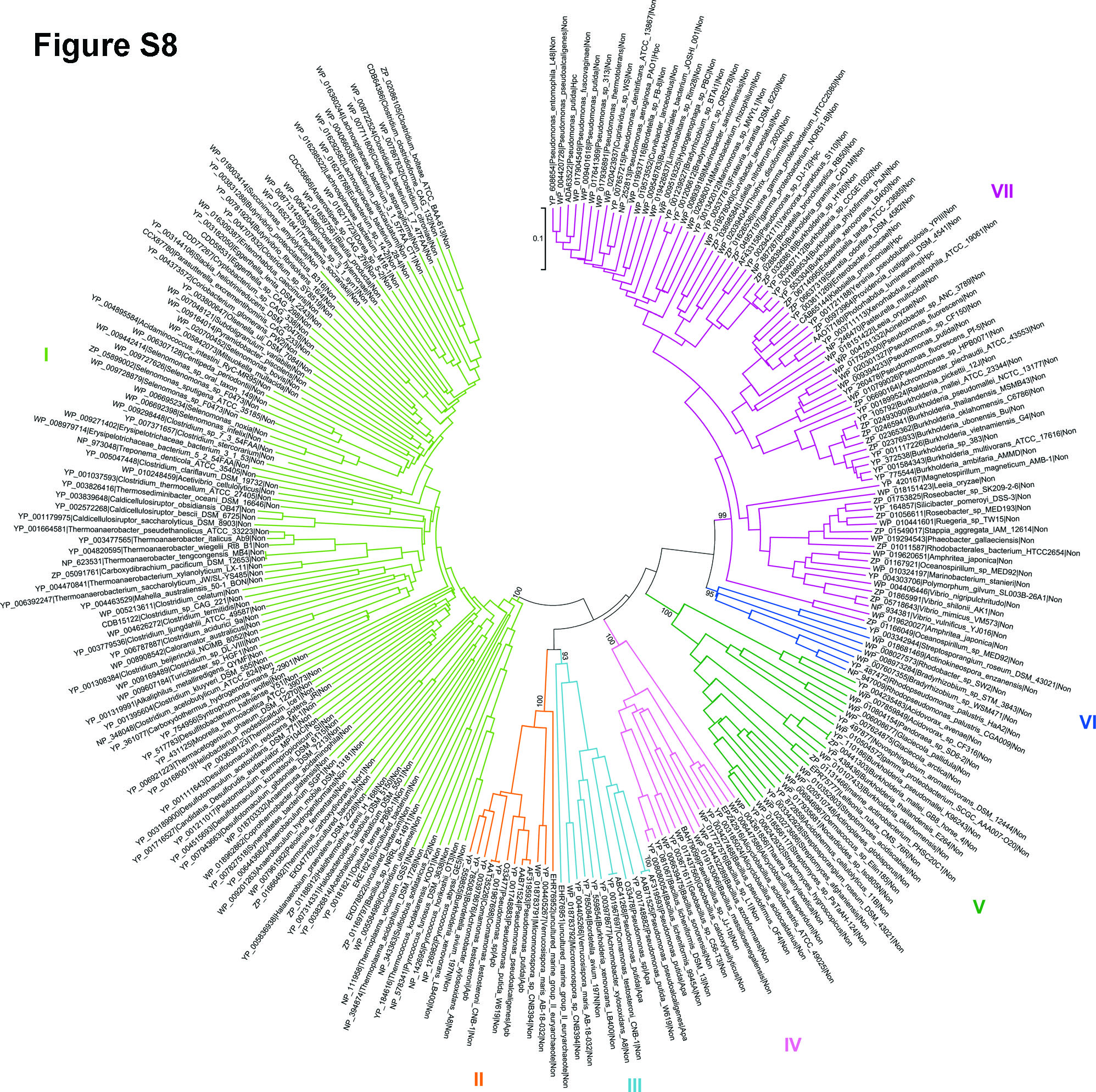

Supplement: Supplementary Data [file supp_bau118_suppl_data.zip › Figure_S8.jpg]

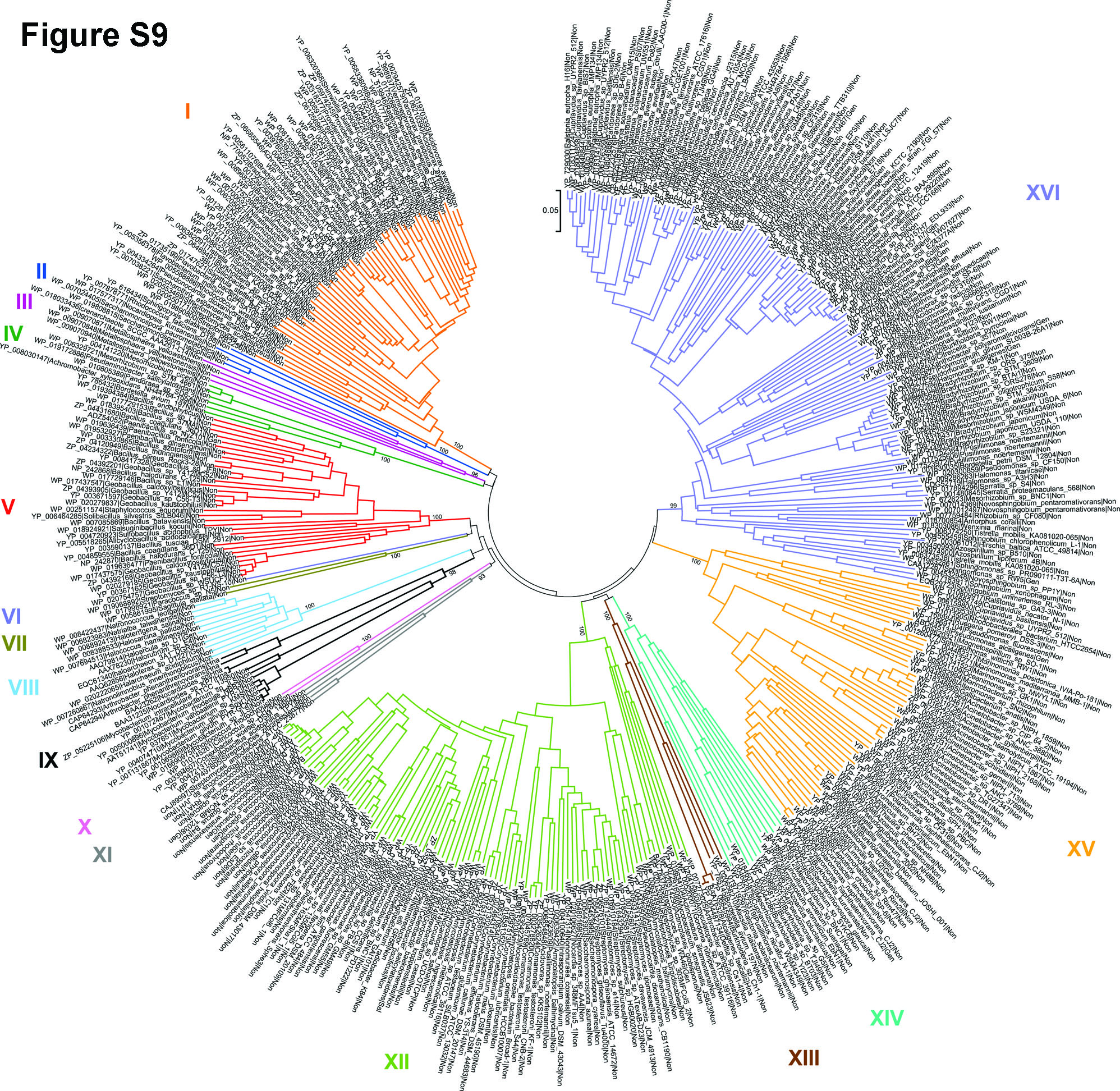

Supplement: Supplementary Data [file supp_bau118_suppl_data.zip › Figure_S9.jpg]

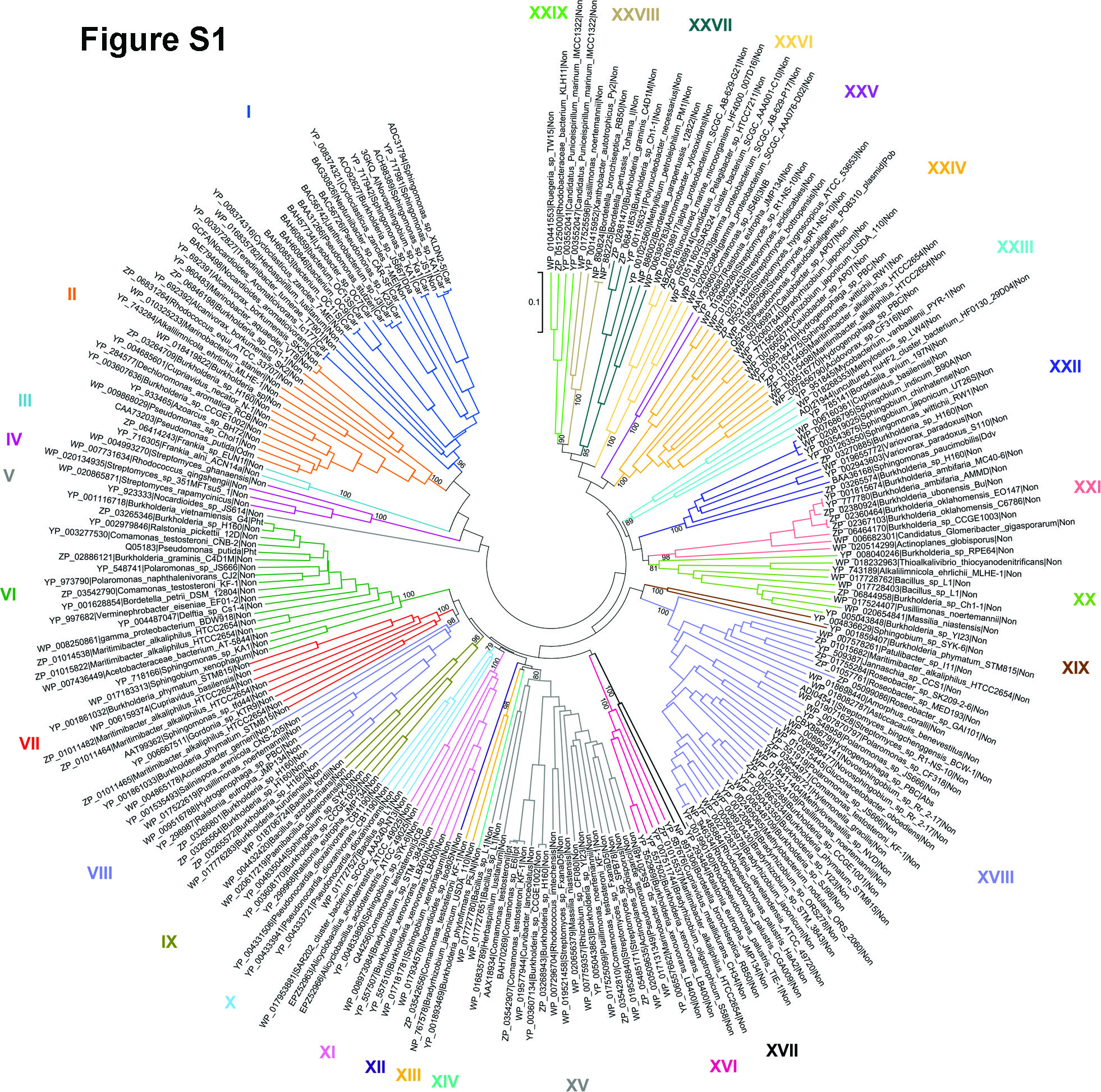

Supplement: Supplementary Data [file supp_bau118_suppl_data.zip › Figure_S1.jpg]

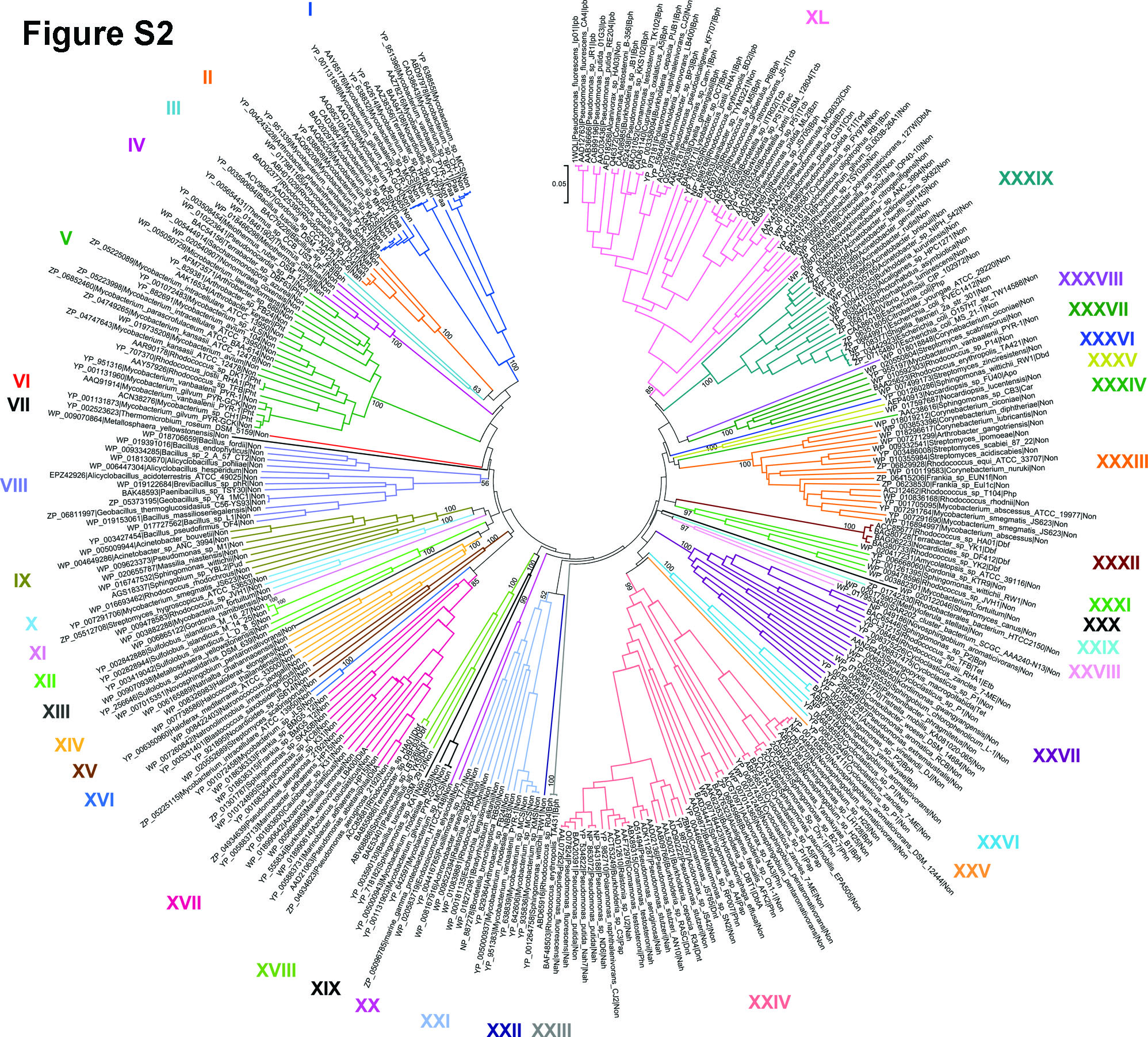

Supplement: Supplementary Data [file supp_bau118_suppl_data.zip › Figure_S2.jpg]

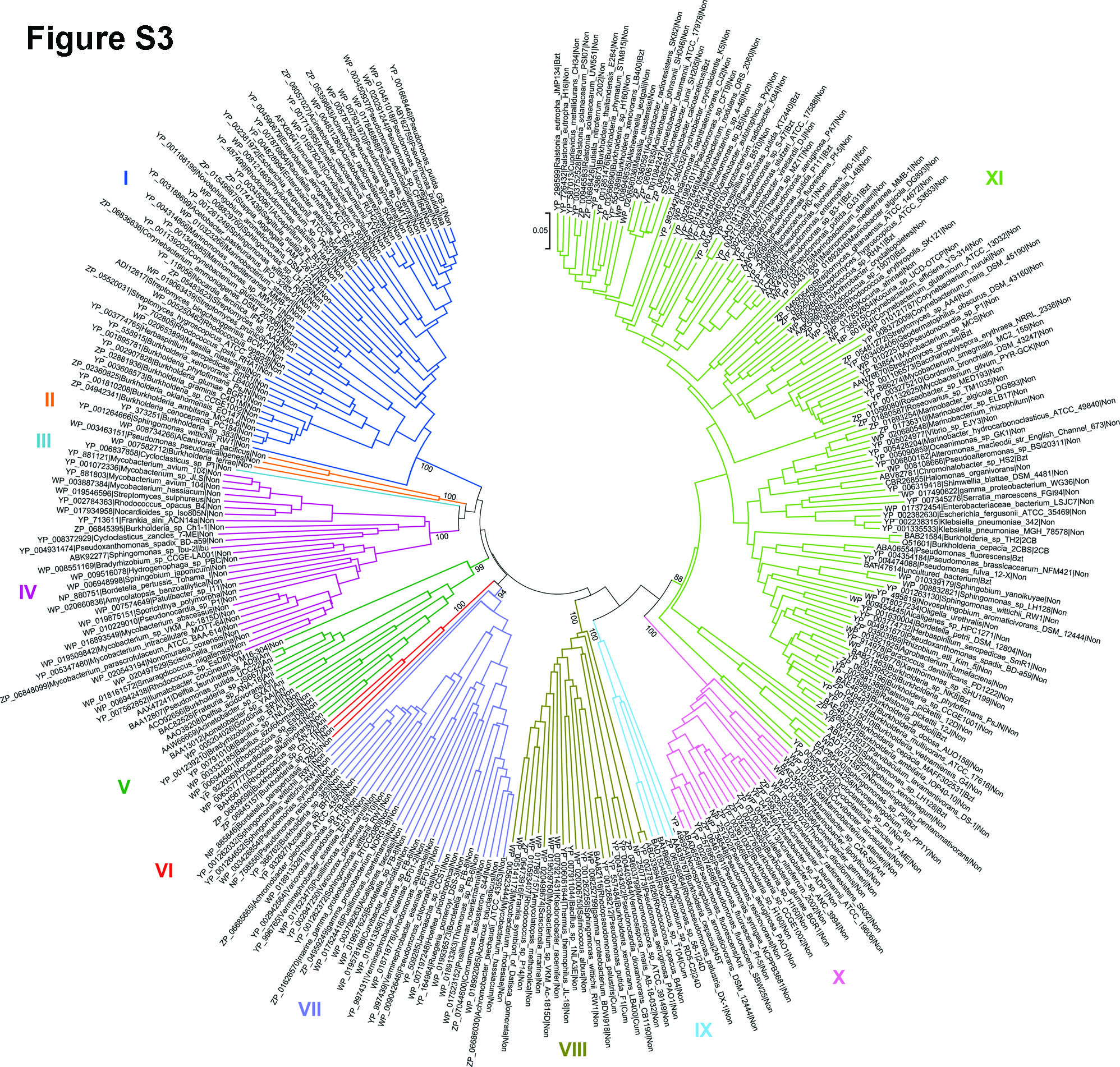

Supplement: Supplementary Data [file supp_bau118_suppl_data.zip › Figure_S3.jpg]

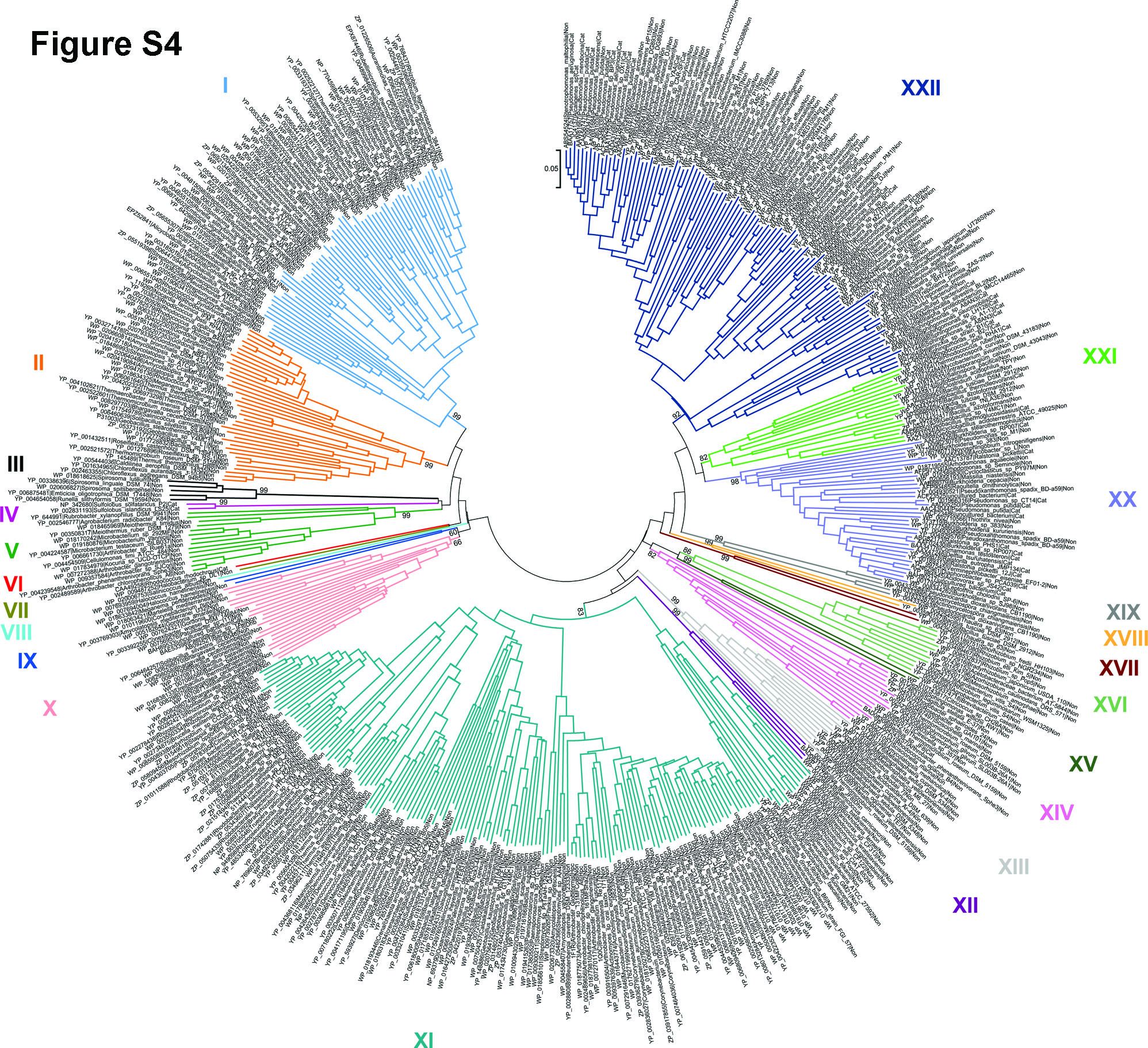

Supplement: Supplementary Data [file supp_bau118_suppl_data.zip › Figure_S4.jpg]

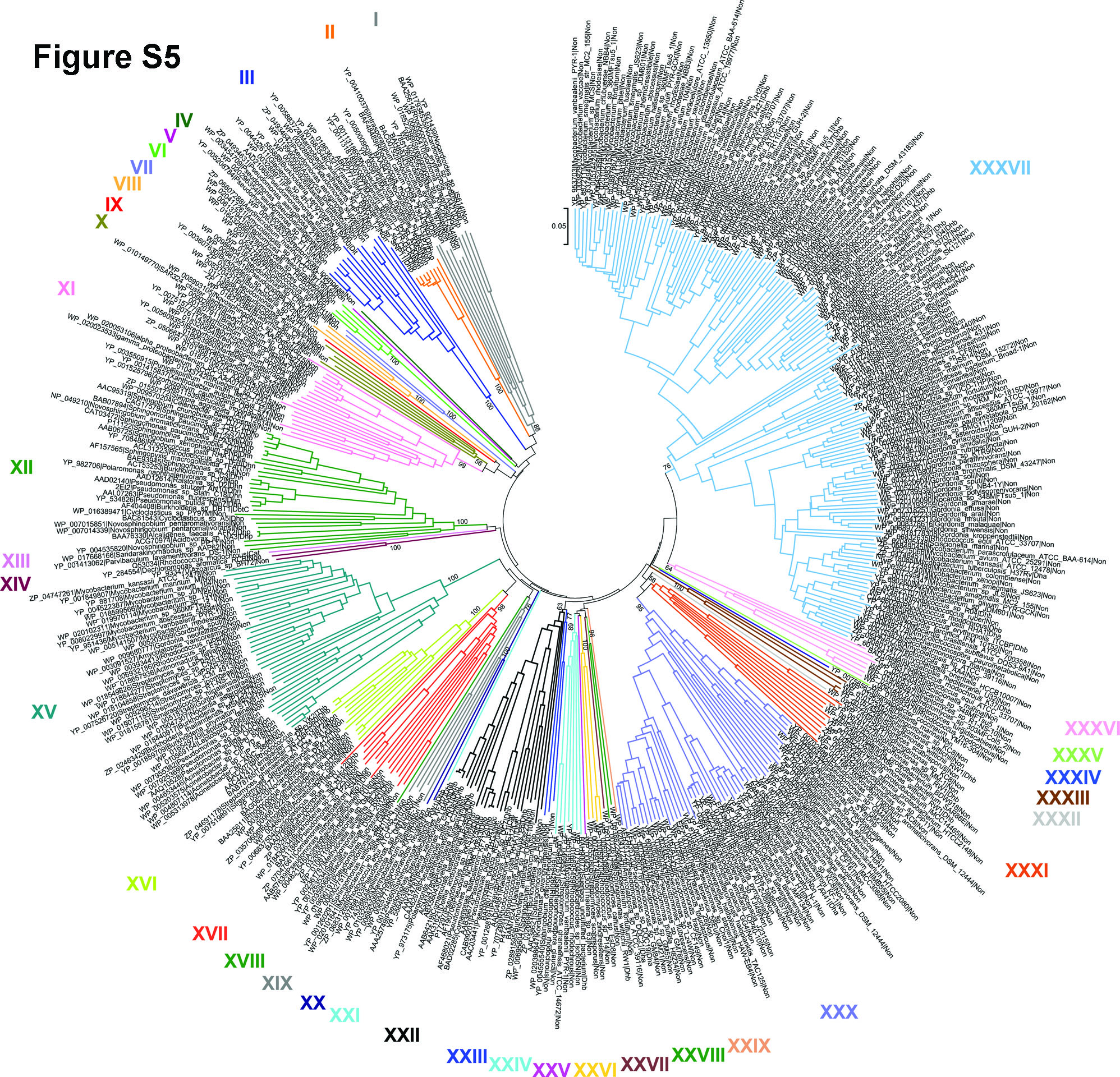

Supplement: Supplementary Data [file supp_bau118_suppl_data.zip › Figure_S5.jpg]
