# Supplementary material for: AromaDeg, a novel database for phylogenomics of aerobic bacterial degradation of aromatics
Source: Database (Oxford). 2014 Dec 1;2014:bau118. doi: 10.1093/database/bau118 (PMC4250580; doi:10.1093/database/bau118)
Supplement: Supplementary Data [file supp_bau118_suppl_data.zip › Legends_to_supplementary_figures.doc]

**Supplementary figure legends**

**Figure S1.** Evolutionary relationships of α-subunits of the phthalate family of Rieske non-heme iron oxygenases. Protein sequences were aligned with MAFFT and the phylogenetic tree was constructed with MEGA5 using the neighbor-joining algorithm with p-distance correction and pairwise deletion of gaps and missing data. A total of 100 bootstrap replications were done to test for branch robustness (bootstrap values are shown adjacent to each cluster node) and redundant protein sequences (> 95 – 99% of sequence identity) were removed. According to the documented substrate specificity of representative members they can be clustered as follows: cluster I – carbazol and diphenylamin dioxygenases; cluster II – 1-Oxo-2,3-dihydroquinoline monooxygenases and related enzymes; clusters III, IV, V, XIX, XX, XXI, XXIII, XXVI, XXVII, XXVIII and XXIX – Rieske oxygenases of unknown function; cluster VI – phthalate 4,5-dioxygenases; clusters VII and VIII –phthalate 4,5-dioxygenases; clusters IX, X, XII, XIII, XIV, XVI and XVII – putative phthalate 4,5-dioxygenases; cluster XI – 3-chlorobenzoate dioxygenases and related enzymes, probable phthalate dioxygenase activity; cluster XV – isophthalate dioxygenases and related enzymes; cluster XVIII – 4-aminobenzenesulfonate 3,4-dioxygenase and related enzymes; cluster XXII – enzymes related to 5,5'-dehydrodivanillate demethylase; cluster XXIV – enzymes related to phenoxybenzoate, function often unknown; cluster XXV – 3-nitrobenzoate dioxygenases and related enzymes. Further information about each cluster is included in additional **Table S1**.

**Figure S2.** Evolutionary relationships of α-subunits of the biphenyl family of Rieske non-heme iron oxygenases. Protein sequences were aligned with MAFFT and the phylogenetic tree was constructed with MEGA5 using the neighbor-joining algorithm with p-distance correction and pairwise deletion of gaps and missing data. A total of 100 bootstrap replications were done to test for branch robustness (bootstrap values are shown adjacent to each cluster node) and redundant protein sequences (> 95 – 99% of sequence identity) were removed. According to the documented substrate specificity of representative members they can be clustered as follows: cluster I – polycyclic aromatic hydrocarbons dioxygenases of Actinobacteria; cluster II – naphthalene dioxygenases of Actinobacteria; cluster III – biphenyl dioxygenases of Bacilli; clusters IV, VI, VII, XI, XV, XIX, XX, XXI and XXII – Rieske oxygenases of unknown function; cluster V – phthalate dioxygenases of Actinobacteria; cluster VIII – Rieske oxygenases of Firmicutes; cluster IX – phenylurea herbicide N-demethylase and related enzymes; clusters X and XIII – Rieske oxygenases of Actinobacteria; clusters XII and XIV – archaeal Rieske oxygenases of unknown function; cluster XVI – Rieske oxygenases of Mycobacteria; cluster XVII – diterpenoid dioxygenases and related enzymes; cluster XVIII – angular dioxygenases of dibenzofuran or fluorene metabolism and related enzymes; cluster XXIII – biphenyl dioxygenases of Actinobacteria; cluster XXIV – naphthalene dioxygenases of Proteobacteria and related enzymes; cluster XXVI – biphenyl dioxygenases of Proteobacteria; cluster XXVII – tetralin, bipheny, ethylbenzene, xylene dioxygenases and related enzymes; cluster XXXII – angular dioxygenases of dibenzofuran metabolism; cluster XXXIII – phenylpropionate dioxygenases of Actinobacteria; cluster XXXIV – carbazol dioxygenases; cluster XXXV – enzymes involved in apoptolidins synthesis; cluster XXXVI – dioxin dioxygenases; cluster XXXIX – phenylpropionate dioxygenases of Proteobacteria; cluster XL – biphenyl/benzene dioxygenaseses. Further information about each cluster is included in additional **Table** **S2**.

**Figure S3.** Evolutionary relationships of α-subunits of the benzoate family of Rieske non-heme iron oxygenases. Protein sequences were aligned with MAFFT and the phylogenetic tree was constructed with MEGA5 using the neighbor-joining algorithm with p-distance correction and pairwise deletion of gaps and missing data. A total of 100 bootstrap replications were done to test for branch robustness (bootstrap values are shown adjacent to each cluster node) and redundant protein sequences (> 95 – 99% of sequence identity) were removed. According to the documented substrate specificity of representative members they can be clustered as follows: cluster I – enzymes involved in indole acetic acid degradation and related enzymes; clusters II, III, VI and VII – Rieske oxygenases of unknown function; cluster IV – ibuprofen-CoA dioxygenases and related enzymes; cluster V – aniline dioxygenases; cluster VIII – p-cumate dioxygenases; cluster IX – 2,4-D and 2,4,5-T oxygenases; cluster X –anthranilate dioxygenases of γ-Proteobacteria and β-Proteobacteria; cluster XI – benzoate and 2-chlorobenzoate dioxygenases. Further information about each cluster is included in additional **Table S3**.

**Figure S4.** Evolutionary relationships of family I.2 proteins (Eltis and Bolin, 1996) of extradiol dioxygenases of the vicinal oxygen chelate superfamily where characterized enzymes typically have a preference for monocyclic substrates. Protein sequences were aligned with MAFFT and the phylogenetic tree was constructed with MEGA5 using the neighbor-joining algorithm with p-distance correction and pairwise deletion of gaps and missing data. A total of 100 bootstrap replications were done to test for branch robustness (bootstrap values are shown adjacent to each cluster node) and redundant protein sequences (> 95 – 99% of sequence identity) were removed. According to the documented substrate specificity of representative members they can be clustered as follows: cluster I, V, VII, VIII, IX, XVII, XVIII and XIX – probable catechol 2,3-dioxygenases; cluster II – enzymes related to catechol 2,3-dioxygenases of *Geobacillus stearothermophilus* FDTP-3; cluster III – probable catechol 2,3-dioxygenases of Bacteroidetes; cluster IV – catechol 2,3-dioxygenases of Archaea; cluster VI – catechol 2,3-dioxygenases; cluster X – enzymes related to 2,3-dihydroxybiphenyl dioxygenases of *Bacillus* sp. JF8; cluster XI – homoprotocatechuate 3,4-dioxygenases (except of β- and γ-Proteobacteria); cluster XII – enzymes related to dihydroxynaphthalene dioxygenase of *Bacillus* sp. JF8; cluster XIII – putative homoprotocatechuate dioxygenases of Archaea; cluster XIV – enzymes related to catechol 2,3-dioxygenase of *Bacillus* sp. JF8; cluster XV – probable catechol 2,3-dioxygenases of γ-Proteobacteria; cluster XVI – probable catechol 2,3-dioxygenases of α-Proteobacteria; cluster XX – proteobacterial catechol 2,3-dioxygenases (EXDO 1.2 D as defined by Eltis and Bolin, 1996); cluster XXI – actinobacterial and Firmicutes catechol 2,3-dioxygenases; cluster XXII – proteobacterial catechol 2,3-dioxygenases (EXDO 1.2 A-C as defined by Eltis and Bolin, 1996). Further information about each cluster is included in additional **Table S4**.

**Figure S5.** Evolutionary relationships of family I.3 proteins (Eltis and Bolin, 1996) of extradiol dioxygenases of the vicinal oxygen chelate superfamily where characterized enzymes typically have a preference for bicyclic substrates. Protein sequences were aligned with MAFFT and the phylogenetic tree was constructed with MEGA5 using the neighbor-joining algorithm with p-distance correction and pairwise deletion of gaps and missing data. A total of 100 bootstrap replications were done to test for branch robustness (bootstrap values are shown adjacent to each cluster node) and redundant protein sequences (> 95 – 99% of sequence identity) were removed. According to the documented substrate specificity of representative members they can be clustered as follows: cluster I –extradiol dioxygenases related to BphC5 of *Rhodococcus erythropolis* TA421; cluster II – extradiol dioxygenases involved in cleavage of dihydroxylated polycyclic aromatic hydrocarbons in Actinobacteria; cluster III – extradiol dioxygenases of diterpenoid degradation; clusters IV, V, VI, VII, VIII, IX, XI, XIV, XIX, XX, XXIV, XXV, XXVI, XXVII, XXIX, XXXII, XXXIV, XXXV – extradiol dioxygenases of unknown function; cluster X – proteobacterial extradiol dioxygenases of unknown function; cluster XII – dihydroxynaphthalene dioxygenases and related enzymes in Proteobacteria; cluster XIII – catechol 2,3-dioxygenases from *Rhodococcus rhodochrous*; cluster XV – enzymes related to extradiol dioxygenases involved in orcinol degradation; cluster XVI – enzymes typically involved in 2,3,2'-trihydroxybiphenyl degradation (e.g. formed from dibenzofuran); cluster XVII – protobacterial 2,3-dihydroxybiphenyl dioxygenases; cluster XVIII – 2,3,2'-trihydroxybiphenyl dioxygenases; cluster XXI – 2,3-dihydroxybiphenyl dioxygenases; cluster XXII – 2,3-dihydroxybiphenyl dioxygenases of Proteobacteria and Actinobacteria; cluster XXIII – 2'-aminobiphenyl-2,3-diol dioxygenases and related enzymes; cluster XXVIII – extradiol dioxygenases related to Swit 0894 DHB dioxygenases of *Sphingomonas wittichii* RW1; cluster XXX – enzymes involved in steroid metabolism by Proteobacteria and related enzymes; some enzymes with dihydroxybiphenyl dioxygenase activity; cluster XXXI – extradiol dioxygenases related to Swit 1826 DHB dioxygenases of *Sphingomonas wittichii* RW1; cluster XXXIII – enzymes related to Swit 1848 DHB dioxygenases of *Sphingomonas wittichii* RW1; cluster XXXVI – extradiol dioxygenases related to BphC4 of *Rhodococcus rhodochrous* K37; cluster XXXVII – enzymes most probably involved in cholesterol and steroid metabolism; some enzymes with dihydroxybiphenyl dioxygenase activity. Further information about each cluster is included in additional **Table S5**.

**Figure S6.** Evolutionary relationships of proteins of a third family of extradiol dioxygenases of the vicinal oxygen chelate superfamily comprising, among others, enzymes using miscellaneous substrates such as 2,3-dihydroxybenzoate. Protein sequences were aligned with MAFFT and the phylogenetic tree was constructed with MEGA5 using the neighbor-joining algorithm with p-distance correction and pairwise deletion of gaps and missing data. A total of 100 bootstrap replications were done to test for branch robustness (bootstrap values are shown adjacent to each cluster node) and redundant protein sequences (> 95 – 99% of sequence identity) were removed. According to the documented substrate specificity of representative members they can be clustered as follows: cluster I – 2,3-dihydroxy-p-cumate dioxygenases and 2,3-dihydroxybenzoate dioxygenases; clusters II and V – putative 2,3-dihydroxybiphenyl dioxygenases; cluster III – 2,3-dihydroxybiphenyl dioxygenases of Firmicutes and Actinobacteria; cluster IV – putative 2,3-dihydroxybiphenyl dioxygenases of Proteobacteria; clusters VI and XVII – actinobacterial extradiol dioxygenases of unknown function; clusters VII and XV – extradiol dioxygenases of unknown function, some enzymes with 2,3-dihydroxybiphenyl cleavage activity; cluster VIII – 2,4,5-trihydroxytoluene dioxygenases and related enzymes; clusters IX, X, XI, XII, XIX, XX and XXI – extradiol dioxygenases of unknown function; cluster XIII – putative extradiol dioxygenases of Leptospira; cluster XIV – 2,3-dihydroxybiphenyl dioxygenases and enzymes of methylhydroquinone metabolism; cluster XVI and XVIII – proteobacterial extradiol dioxygenases of unknown function. Further information about each cluster is included in additional **Table S6**.

**Figure S7.** Evolutionary relationships of proteins of the protocatechuate family of the LigB superfamily extradiol dioxygenases. Protein sequences were aligned with MAFFT and the phylogenetic tree was constructed with MEGA5 using the neighbor-joining algorithm with p-distance correction and pairwise deletion of gaps and missing data. A total of 100 bootstrap replications were done to test for branch robustness (bootstrap values are shown adjacent to each cluster node) and redundant protein sequences (> 95 – 99% of sequence identity) were removed. According to the documented substrate specificity of representative members they can be clustered as follows: cluster I – protocatechuate dioxygenases; clusters II, III and IV – putative protocatechuate dioxygenases; cluster V – gallate dioxygenases; clusters VI, VII, IX, X, XI and XIII – extradiol dioxygenases of unknown function; cluster VIII – extradiol dioxygenases involved in metabolism of 2'-substituted 2,3-dihydroxibiphenyls; cluster XII – extradiol dioxygenases related to PhnC probably transforming polycyclic arene diols; cluster XIV – extradiol dioxygenases related to those involved in 3-hydroxy-4-pyridone degradation; clusters XV and XVIII – archaeal extradiol dioxygenases of unknown function; clusters XVI, XXIII, XXIV and XXV – actinobacterial extradiol dioxygenases of unknown function; cluster XVII – 2,2-Dihydroxy-p-cumate dioxygenase of Actinobacteria and related enzymes; cluster XIX – extradiol dioxygenases of unknown function of Mycobacteria; cluster XX – extradiol dioxygenases possibly involved in fluorene degradation; cluster XXI – 3-O-methylgallate 3,4-dioxygenase DesZ; cluster XXII – aminobiphenyl-2,3-diol 1,2-dioxygenases; cluster XXVI – 3-sulfocatechol dioxygenases; cluster XXVII – 2,3-dihydroxyphenylpropionate dioxygenases; cluster XXVIII – probable 2,3-dihydroxyphenylpropionate dioxygenases; clusters XXIX, XXX and XXXI – 2,3-dihydroxyphenylpropionate dioxygenases; cluster XXXII – putative 2,3-dihydroxyphenylpropionate dioxygenases. Further information about each cluster is included in additional **Table S7**.

**Figure S8.** Evolutionary relationships of proteins of the homoprotocatechuate family of the LigB superfamily extradiol dioxygenases. Protein sequences were aligned with MAFFT and the phylogenetic tree was constructed with MEGA5 using the neighbor-joining algorithm with p-distance correction and pairwise deletion of gaps and missing data. A total of 100 bootstrap replications were done to test for branch robustness (bootstrap values are shown adjacent to each cluster node) and redundant protein sequences (> 95 – 99% of sequence identity) were removed. According to the documented substrate specificity of representative members they can be clustered as follows: cluster I – extradiol dioxygenases of unknown function, mainly observed in Clostridia and Archaea; cluster II – 2-aminophenol 1,6-dioxygenase beta subunit; cluster III – 2-aminophenol 1,6-dioxygenase alpha subunit; clusters IV, V and VI – extradiol dioxygenases of unknown function; cluster VII – homoprotocatechuate 2,3-dioxygenases. Further information about each cluster is included in additional **Table S8**.

**Figure S9.** Evolutionary relationships of proteins of the gentisate family of the cupin superfamily extradiol dioxygenases. Protein sequences were aligned with MAFFT and the phylogenetic tree was constructed with MEGA5 using the neighbor-joining algorithm with p-distance correction and pairwise deletion of gaps and missing data. A total of 100 bootstrap replications were done to test for branch robustness (bootstrap values are shown adjacent to each cluster node) and redundant protein sequences (> 95 – 99% of sequence identity) were removed. According to the documented substrate specificity of representative members they can be clustered as follows: cluster I – gentisate dioxygenases; clusters II, III, IV, VI, VII, X, XI and XIV – putative gentisate dioxygenases; cluster V – gentisate dioxygenases of Firmicutes; cluster VIII – gentisate dioxygenases of Archaea; cluster IX – 1-hydroxy-2-naphthoate dioxygenases of Actinobacteria; cluster XII – gentisate dioxygenases of Actinobacteria and Proteobacteria; cluster XIII – 1-hydroxy-2-naphthoate dioxygenases of Proteobacteria; clusters XV and XVI – gentisate dioxygenases of Proteobacteria. Further information about each cluster is included in additional **Table S9**.
